# Nebulized budesonide combined with systemic corticosteroid vs systemic corticosteroid alone in acute severe asthma managed in the emergency department: a randomized controlled trial

**DOI:** 10.1186/s12873-022-00691-9

**Published:** 2022-07-23

**Authors:** Soudani Marghli, Chafiaa Bouhamed, Amira Sghaier, Nabil Chebbi, Insaf Dlala, Samia Bettout, Achref Belkacem, Sarra Kbaier, Nahla Jerbi, Abdelouahab Bellou

**Affiliations:** 1 Faculty of Medicine of Monastir, University of Monastir, 5019 Monastir, Tunisia; 2 Emergency Department, Research Unit «Douleur thoracique», UR17SP09, Tahar Sfar University Hospital, 5100 Mahdia, Tunisia; 3 Faculty of Medicine of Sousse, University of Sousse, 4002 Sousse, Tunisia; 4 Global Health Care Network Research Innovation Institute, LLC, Brookline, MA USA; 5 International Board of Medicine and Surgery, Tampa, Florida USA

**Keywords:** Acute asthma, Nebulized budesonide, Systemic steroid, Emergency department

## Abstract

**Background:**

The additive benefit of inhaled corticosteroid when used with systemic corticosteroid in acute asthma is still unclear. The objective of this study was to assess the effect of high and repeated doses of inhaled budesonide when combined with the standard treatment of adult acute asthma.

**Methods:**

It was a prospective double-blind randomized controlled study performed in the emergency department (ED) from May 1, 2010 to February 28, 2011 (ClinicalTrials.gov, NCT04016220). Fifty patients were included and were randomized to receive intravenous hydrocortisone hemisuccinate in association with nebulized budesonide (*n* = 23, budesonide group) or normal saline (*n* = 27, control group). Nebulization of budesonide or saline was done in combination with 5 mg of terbutaline every 20 min the first hour, then at 2 h (H2), and 3 h (H3). All patients received standard treatment. Efficacy and safety of inhaled budesonide were evaluated every 30 min for 180 min.

**Results:**

A significant increase in peak expiratory flow (PEF) was observed in both treatment groups at evaluation times. The increase in PEF persisted significantly compared to the previous measurement in both groups. There was no significant difference in the PEF between the two groups at evaluation times. There was no significant difference between the two groups in the evolution in the respiratory rate and heart rate. There was also no statistically significant difference between the two groups in the rate of hospitalization, the discharge criteria before the end of the protocol.

**Conclusions:**

Considering its limited power, our study suggests that the association of nebulized budesonide with hydrocortisone hemisuccinate has no additional effect over the use of hydrocortisone alone in adults’ acute asthma managed in the ED.

**Supplementary Information:**

The online version contains supplementary material available at 10.1186/s12873-022-00691-9.

## Background

Inhaled short-acting bronchodilators (short-acting ß_2_-agonists alone or in combination with anti-cholinergic drugs), systemic corticosteroids (SCS), and adequate oxygenation are the basis of the treatment of moderate -to- severe asthma in the emergency department (ED) [1, 2]. Although, SCS represent the standard of care for severe asthma exacerbation in the ED, it is well known that SCS require 6 to 24 hours to produce effects on pulmonary function or reduction of hospitalization [3–5]. Recently a renewed focus on the use of inhaled corticosteroid (ICS) in acute asthma was observed [3–12]. Several studies have shown an effect 90 to 120minutes of ICS on pulmonary function in the treatment of adult [6, 7] and pediatric [8–12] acute asthma. This effect suggests a local non-genomic mechanism, related to a vasoconstriction of smooth muscle on mucosal vessels causing a blood flow reduction in the airway mucosa [13–15]. A published systematic review on the effectiveness of ICS in the ED treatment of acute asthma [16] concluded that there is a beneficial effect compared to placebo. But studies, all in children, comparing ICS to SCS showed contradictory results [11, 17–20]. In the ED setting; there is insufficient evidence that ICS therapy alone can be used with confidence instead of SCS. The additive benefit of ICS when used with SCS is still under investigation and there is insufficient evidence that ICS significantly change pulmonary function or clinical scores in addition to SCS in acute asthma [16]. The objective of our clinical trial was to compare the additive effect of high and repeated doses of inhaled budesonide combined to the standard treatment in adult acute asthma. Our primary hypothesis was that the association of ICS to SCS would be more effective than SCS alone in improving pulmonary function.

## Methods

### Patients

We recruited adult patients with acute asthma who were managed in the ED of Tahar Sfar Hospital in Mahdia, Tunisia from May 1, 2010 to February 28, 2011. The inclusion criteria were: diagnosis criteria of asthma of the Global Strategy for Asthma Management and Prevention [21]; age between 18 and 50 years; a peak expiratory flow (PEF) rate less than 50% of predicted value; one or more of the following features were present: accessory muscle activity, a heart rate greater than 110 beats/minute, a respiratory rate greater than 25 breaths/minute, a limited ability to speak; and an expressed willingness to participate in the study, with written informed consent obtained. Patients were excluded if they had a temperature higher than 38°C, or a history of cardiac, hepatic, renal, or other medical disease, or if they were pregnant. The study was recorded in ClinicalTrial.gov registry under the number: NCT04016220 on 11/07/2019. The local institutional ethics committee (Comité d’Ethique de l’hôpital Tahar Sfar, Mahdia, Tunisia) approved the study. Written, informed consent was obtained from all participants.

### Design

This study was a prospective, randomized, double-blind, controlled trial.

**Fig. 1:**
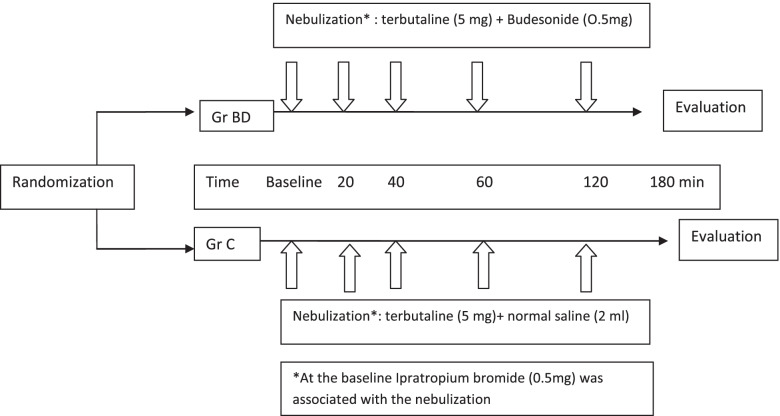
Study protocol. GrBD: budesonide group, GrC: control group, HCHS: hydrocortisone hemisuccinate, i.v.: intravenously

Inclusion of patients in the study was ensured during the day and on working days only. Included patients received the assigned treatment after randomization and contained in sealed envelopes and that neither the treating physician nor the patient knows the nature of the product. Randomization was performed using the GraphPadStateMate® software. Only the principal investigator prepared the drug or placebo in syringes after the envelopes were opened. Budesonide and placebo were identical in appearance and neither the clinician nor the patient can distinguish them. The principal investigator was not involved in the enrolment of participants, assignment to interventions, therapeutic care, and statistical analysis. Patients in the experimental group received a first nebulization of 5 mg of terbutaline (Bricanyl®, AstraZeneca: 5mg/2ml solution) in combination with 0.5 mg of ipratropium bromide (Ipratropium®, AGUETTANT: 0.5 mg/2 ml solution) and 0.5 mg of budesonide (Pulmicort®, AstraZeneca, 0.5 mg/2 ml solution) followed by frequent nebulization of a combination of terbutaline (5mg) and budesonide (0.5mg) at 20, 40, 60 and 120 min. Patients in the control group received a first nebulization of 5 mg of terbutaline (Bricanyl®, AstraZeneca: 5mg/2ml solution) in combination with 0.5 mg of ipratropium bromide (Ipratropium®, AGUETTANT: 0.5 mg/2 ml solution) and placebo (normal saline: 2 ml) followed by frequent nebulization of a combination of terbutaline (5mg) and placebo at 20, 40, 60 and 120 min. All patients received hydrocortisone hemisuccinate (100 mg IV) and oxygen at a flow rate of 7 L/min as a carrier gas for nebulization (Fig. 1).

### Measures

The parameters collected concerned the demographic characteristics, the severity of asthma (background treatment, previous hospitalization and history of mechanical ventilation), the duration of symptoms before presentation, which specifically included how long the patient had been wheezing and shorter of breath than usual, a decline in the PEF, if available, was considered, and the clinical criteria of severity.

The following variables were measured and calculated for each patient immediately before treatment and then every 30 minutes for 3 hours after treatment initiation: PEF, respiratory rate, involvement of the accessory respiratory muscles, dyspnea, heart rate, blood pressure. The PEF was measured by a peak-flow meter (MicroPeaK® Cardinal Health, UK); the highest of 3 successive measurements was used. Dyspnea was assessed by the patient’s own sense of difficulty breathing and a value was assigned on a scale of 0 to 3; 0: absent, 1: minimal, 2: moderate, 3: severe. The clinical aggravation requiring the use of mechanical ventilation led the interruption of the protocol. At the end of the protocol the following side effects were sought by the patient’s interrogation: palpitations, tremor, anxiety, headache, and dry mouth. The decision to release or to admit the patient was made at the end of the protocol (180 minutes) by the senior physician without knowledge of the patient’s treatment group assignment. It requires the meeting of all the following criteria: no involvement of the sternocleidomastoid muscles, the sibilant rails were considered minimal or absent, free of dyspnea, the PEF > 60% of the predicted [8]. At the end of the emergency department, patients were treated with prednisone 40mg/ day for 7 days, short-acting beta-mimetics and an appointment at the pneumology outpatient consultation after 1 week.

Primary outcome measures were defined as follow: improvement in PEF and reduction in admission rate. Secondary outcomes were clinical measures, respiratory and heart rates, side effects, and proportion of patients that reached the discharge threshold during the 3 hours of treatment for each group.

### Statistical analysis

Continuous variables were expressed as mean ± standard deviation (SD) or as median (25–75% interquartile range, IQR) as appropriate while verifying the normality with a Kolmogorov-Smirnov test. Categorical variables were summarized using numbers and percentages. Baseline data of the two treatments were compared using Student’s *t* test for normally distributed independent samples, or the Mann-Whitney U test for no normally distributed continuous variables. Fisher’s exact test was used for categorical variables. A *p* value of less than0.05 using a two-tailed test was taken as significant for all statistical tests. Changes in PEF, HR, RR, and dyspnea scale were evaluated using repeated measures analysis of variance (ANOVA), with one between-subject factor (budesonide-placebo groups) and one within-subject factor (time). One-way repeated measures ANOVA was used to compare baseline values for each variable. At the beginning of the study we determined the sample size needed to detect a 20 L/min difference in PEF variation between groups, the calculations were based in a previous study which showed a variation between baseline and 40 min evaluation was 65 ± 38 l/min [22]. Statistical power calculation showed that 58 subjects per group was needed with α = 0.05 and β = 0.20 (ie, with 80% power). Due to the poor recruitment of patients, the study was stopped.

## Results

### Description of study cohort

**Fig. 2:**
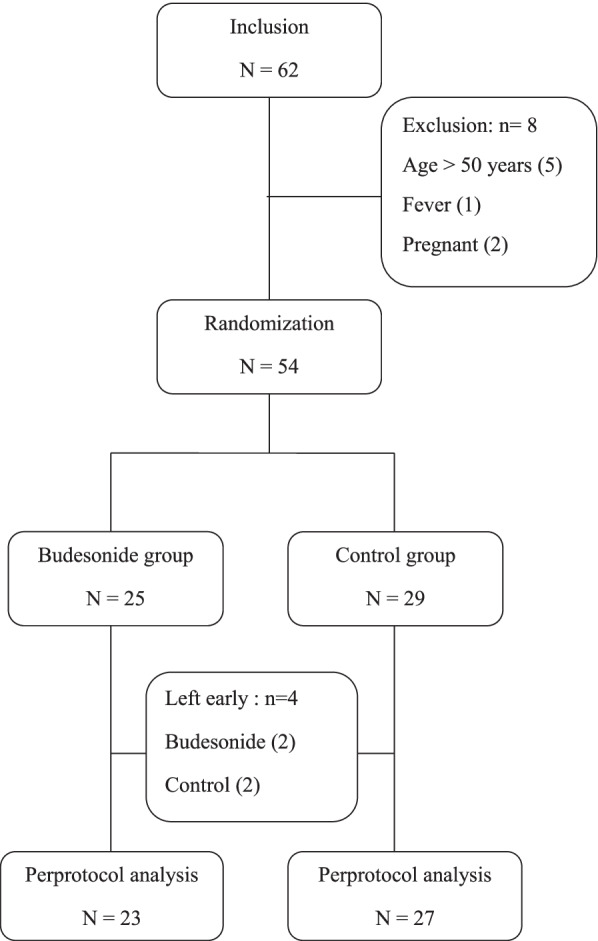
Trial Flowchar

During the study period 62 patients were initially included. Eight patients were excluded, five patients were over 50 years old, one patient had a fever and two patients were pregnant. The remaining 54 patients were randomized into 25 patients in the budesonide group and 29 in the control group. During the protocol two patients from each group, did not complete the protocol and left the ED prematurely (Fig. 2). The analysis consisted of 23 patients in the budesonide group and 27 patients in the control group. Acute asthma was inaugural in 10% of patients (*n* = 5) and 54% of patients (*n* = 27) had an asthma lasting less than or equal to 5 years. The demographic characteristics and the severity of asthma were comparable in both groups (Table 1). Both groups were comparable at inclusion (Table 2).

**Table 1:** Characteristics of included patients in the trial

	Budesonide ***N*** = 23	Control ***N*** = 27	***p***
Age, y, mean (SD)	38 ± 10	33 ± 11	0.086
Sex M	9(39)	11(41)	0.908
Long-term treatment			
Long acting beta_2_-mimetics	9(39)	7(26)	0.318
Inhaled corticosteroids	11(48)	14(52)	O.777
Systemic corticosteroids	1(4)	0	
Theophylline	4(17)	1(4)	0.167
History of hospitalization for acute asthma	5 (22)	8(30)	0.526
1 hospitalization	2	4	
2 hospitalizations	1	4	
3 hospitalizations	1	0	
History of mechanical ventilation for acute asthma	2(9)	2(9)	0.898

All data except age are presented as number (percentage)

**Table 2:** Baseline characteristics at inclusion in the Emergency Department

	Budesonide ***N*** = 23	Control ***N*** = 27	***p***
β2 used within past 48 h	14 (61)	13(48)	0.368
Corticosteroids used within past 7 days	1/21(5)	5/25(19)	0.054
Duration of the crisis, h	35 ± 46	49 ± 80	0.474
Respiratory rate, breaths /min	30 ± 6	29 ± 9	0.636
SatO2, %	94 ± 5	95 ± 4	0.548
PEF, L/min	153 ± 69	163 ± 61	0.588
PEF, % of the predicted	31 ± 14	33 ± 12	0.453
PEF ≤ 30% of the predicted	6/19(32)	8/26(31)	0.954
Systolic arterial blood pressure, mmHg	121 ± 15	120 ± 17	0.578
Diastolic arterial blood pressure, mmHg	70 ± 11	73 ± 11	0.531
Heart rate, beats/min	99 ± 19	92 ± 26	0.339

All data are presented as number (percentage) or mean (±SD)

*β2* short-acting beta_2_-mimetics, *h* hour, *PEF* Peak expiratory flow

### Evaluation of treatment effects

#### PEF

**Fig. 3:**
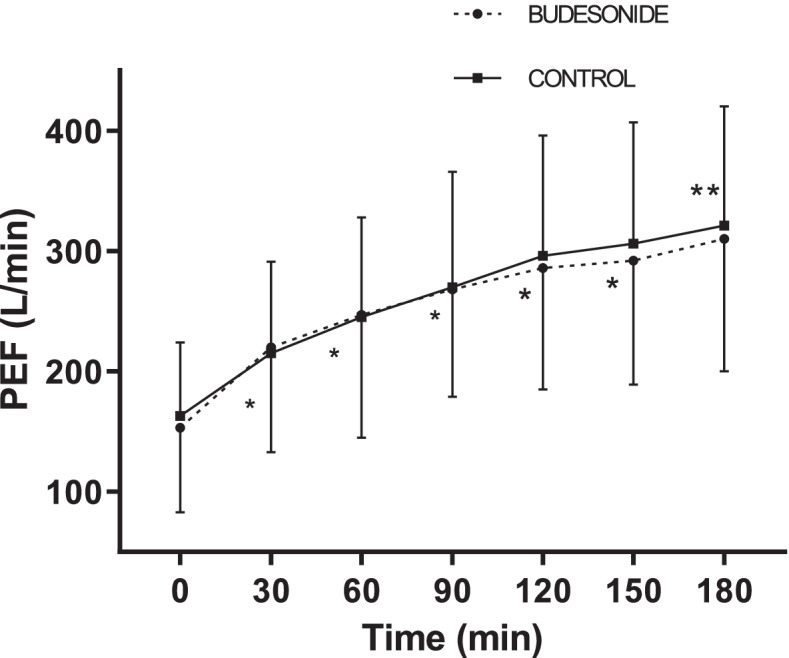
Evolution of PEF. *Values in both groups were significantly better than previous evaluation. ** *p* < 0.05 vs T150 in control group. PEF: peak expiratory flow

The PEF increased significantly at the different evaluation times compared to the previous evaluation times until 150 min for the budesonide, whereas this statistical significance continues until 180 min in the control group (Fig. 3). The average PEF at 180 min was 308 ± 107 l/min and 321 ± 99 l/min respectively in the budesonide (*n* = 22) and control (*n* = 27) groups. The increase in PEF at 180 min was 139 and 121% respectively for the budesonide group and control with a difference of 18% (95% CI [− 62 to 98%]). We did not find statistically significant difference in the PEF between the two groups at the different evaluation times.

### RR

We found that the decrease in RR was statistically significant compared to baseline only from 60 min for both groups (see Additional Figure 1). There was no statistically significant difference in RR at the different evaluation times between the two groups.

### Dyspnea scale

The decrease in dyspnea scale was no longer significant compared to the previous evaluation time from 90 min in both groups. There was no statistically significant difference in the dyspnea scale between the two groups (see Additional Figure 2).

### HR

There was a tendency for an increase in HR compared to the baseline in the control group, which became statistically significant at 120 and 180 min, while the variation was not statistically significant in the budesonide group (see Additional Figure 3). HR tends to be higher in the control group compared to the budesonide group only at 180 min (102 ± 17 beats / min in the control group vs 94 ± 17 beats / min in the budesonide group, *p* = 0.096).

### Outcome and side effects

The hospitalization rate was 35 and 33% in the budesonide and control groups, respectively, without significant difference (Table 3). In intent-to-treat analysis, there was no statistically significant difference between the two groups, 40% in budesonide group vs 38% in control group (*p* = 0.876). Similarly, the proportion of patients with discharge criteria before the end of the protocol (180 min) and the incidence of side effects were comparable between the two groups.

**Table 3:** Outcomes and side effects

	Budesonide ***N*** = 23	Control ***N*** = 27	Absolute risk reduction (95% CI)
Hospitalization, n(%)	8(35)	9(33)	−2(−28 to 24)
Discharge criteria before 180 min, n (%)	10(44)	12(44)	−0.9(−28.6 to 26.7)
Side effects, n (%)	8(35)	9(33)	−2(−28 to 24)
Palpitation, n	6	6	
Tremor, n	5	3	
Headache, n	2	2	
Dry mouth, n	2	4	

All data are presented as number (percentage)

### Evaluation in the most severe patients

Fifteen patients had a PEF at baseline of less than 30% of the predicted value. The clinical characteristics of severity of the asthma were comparable between the two groups (see Additional Table 1). PEF increased significantly at the different evaluation times compared to the baseline and there was no statistically significant difference in the PEF between the two groups at the different time periods. However, this gain in PEF is no longer significant compared to the previous evaluation times from 60 min and 90 min for budesonide and control groups, respectively. The increase in PEF at 180 min was 182% ± 83% in the budesonide group and 149% ± 93% fin the control group (*p* = 0.529). There was no statistically significant difference between the two groups in the decrease of RR, dyspnea scale, and HR. Hospitalization rate was 67 and 33% in the budesonide group and control group respectively, without reaching a statistically significant difference. There was no statistically significant difference between the two groups in discharge criteria and incidence of side effects (see Additional Table 2).

## Discussion

The objective of this randomized controlled double-blind trial was to compare the efficacy and tolerability of the combination of repeated nebulization of budesonide with HCHS i.v. versus HCHS i .v. alone in severe acute asthma in adults managed in the ED. The results of the trial did not find a statistically significant difference between the two groups in the rate of hospitalization, the discharge criteria before the end of the protocol, the improvement of PEF, and the decrease in RR. There was also no statistically significant difference between the two groups with regard to decreased of dyspnea. HR was higher in the control group at the end of the protocol. Finally, there was no difference in the rates of side effects between the two treatment groups.

We have excluded patients over the age of 50 because the increased incidence of morbidities and in the context of the emergency, it is sometimes difficult to eliminate other pathologies such as chronic obstructive pulmonary disease. Probably for this reason that Rodrigo G [4, 7, 8] also included patients between the ages 18 to 50 in his work evaluating inhaled corticosteroids therapy in the emergency department on patients with acute asthma. On the other hand, only 48–52% of the study participants used inhaled corticosteroids in the baseline before emergency department admission because this drug is relatively expensive. In a study comparing the effectiveness of nebulized salbutamol and adrenaline in acute severe asthma in ICU, inhaled corticosteroids were used at 0 and 18% [22]. Furthermore, it is not likely that the results are influenced by the severity of the patients in the budesonide group since 4% of the patients were under systemic corticosteroids because there is no significant difference between groups at inclusion and even in the sub-group analysis for the more severely affected participants there was no significant difference in outcomes between groups.

SCS associated with repeated nebulization of mimetic beta2, anticholinergics and adequate oxygenation are the basis of the treatment for severe acute asthma in the ED [3, 4]. In addition to the delayed anti-inflammatory effect of corticosteroids which happens within a few hours or days, the acute therapeutic response of ICS indicates a different mechanism of action of topical character (nongenomic action) [13]. Evidence suggests that ICS decreases airway blood flow by modulating sympathetic nervous system control through potentiating vasoconstriction by increased noradrenergic neurotransmission in the airway vessels [15, 23]. This effect is transient, it reaches its peak effectiveness in 30 minutes then returns to basal value between 60 to 90 min [24]. In addition, the decrease in airway blood flow is likely to increase the action of inhaled bronchodilators by diminishing their clearance from the airway [25]. Thus, simultaneous administration of ICS and bronchodilators may have a rapid clinical effect. New data on the non-genomic mechanism of action of ICS have opened in recent years a new pathway on the treatment of acute asthma. The first placebo-controlled trials in adults [6, 7] and in children [9–11] were favorable for the use of ICS in acute asthma, showing an improvement in physiological parameters. The combination of 12 studies in a meta-analysis published in 2012 including 960 patients indicates a significant reduction in hospital admissions in patients with ICS than placebo [16]. Approximately eight patients would require ICS treatment to prevent one admission.

The benefit effect in reducing hospital admissions persist in the subgroup ICS plus SCS than SCS alone, however there was heterogeneity between studies [16]. Other studies comparing ICS to SCS showed contradictory results [11, 17–20, 26–28]. In six studies including children with moderate acute asthma, ICS were comparable to SCS regarding improvement in physiological parameters with a tendency to have an earlier beneficial effect [11, 17–19, 26, 27]. In more severe children, Schuh et al. [29] found a greater improvement in the maximum expiratory volume per second (18.9% vs 9.4%) and a reduction in the hospitalization rate (10% vs 31%) in the group receiving prednisone (2 mg / kg tablet) than in the inhaled fluticasone group (single dose of 2 mg). In contrast, in the single adult acute asthma trial comparing the effects of repeated fluticasone inhalation versus hydrocortisone i.v., Rodrigo found early improvement in physiological parameters in the fluticasone group [30]. The benefit was more evident in the more severe patients (FEV_1_ < 1L) with a decrease in hospitalizations (38% in the fluticasone group vs 25% in the hydrocortisone group, *p* = 0.05). The inclusion of these studies in the meta-analysis cited above did not find a clear advantage for either ICS or SCS in terms of hospital admissions [16] but there was significant heterogeneity between studies. The authors of this meta-analysis suggested further investigations in order to rule out the sources of heterogeneity due to the limited number of studies included and the limitation of the judgment criteria. Nevertheless, these comparative studies have had the additional advantage of explaining the mechanism of action of corticosteroids. In two studies, it was found a reduction in the number of eosinophils in the blood after use of prednisone and a reduction in the number of eosinophils in the airways after use of fluticasone suggesting a local anti-inflammatory effect of ICS [31].

Taken together, these results reinforced the choice of using ICS in association with SCS in our study. Probably the ICS cannot control all the inflammatory mechanisms encountered in asthma. The determination of the additional benefits of the association of ICS with the usual treatment in acute asthma in adults has not been extensively studied. Only few randomized controlled trials have been published to date [32–35]. In one study that included adults with a methodology similar to our RCTs, Guttman et al. included 60 acute asthma patients in the ED [32]. All patients received a nebulization of salbutamol (base, 30 min, 1h, 2h, 4h, 6h, 8h and 10h) and 80mg of methylprednisolone at i.v. at the inclusion and 40mg after 6 hours. After randomization, patients in the experimental group received 7mg over 8 hours (base, 30 min, 1 h, 2h, 4h, 6h and 8h) of beclomethasonedipropionate (BDP) through a metered dose inhaler. A placebo was administrated in the same way in the control group. After initial treatment, a significant increase in FEV1, predicted FEV and PEF percentage change (*p* < 0.001) was associated with a decrease in dyspnea score and RR (*p* < 0.03) in the two groups. At 120 minutes, a plateau was reached and only minor changes were observed thereafter. Variations in spirometry, dyspnea score, and vital parameters did not differ between the two groups at the different evaluation times. Authors concluded that the addition of BDP to standard therapy (methylprednisolone IV and β agonist) has no additional benefits over standard treatment. In this study, the PEF evolution in each group was similar whereas in our study the gain of the PEF was no longer significant from 150 min compared to the previous evaluation time for the budesonide group while it persisted significant until the end of the protocol in the control group. We suspect that our patients were more severe than those in Guttman et al. probably, the lower the PEF is the better the response to the combination of ICS plus SCS. We did not confirm our hypothesis in subgroup of patients (*n* = 15) with a PEF < 30% of the predicted value of our trial. A trial involving a larger number of more severe patients could answer this question. The second explanation is the time interval of administration of ICS which was 20 min for the first four administrations in our protocol and which was more prolonged in Guttman et al. study with 30 min for the first two administrations and 60 min later. In fact, it has been demonstrated that the non-genomic effect of ICS is transient with a maximum of 30–60 min after administration [24] and it could be of interest to administer repeatedly at time intervals of less than 30 mi n[25]. Finally, fluticasone and budesonide have been shown to produce more effect than BDP [36]. In another study Bateman et al. [33] compared the efficacy and safety of budesonide / formoterol with formoterol alone in 115 adult asthma patients who showed evidence of refractoriness to a short-acting β_2_-agonist. They have shown a similarly rapid relief of acute bronchoconstriction in the two treatment groups and there were no statistically significant difference between-groups for treatment success and treatment failure. In a pediatric randomized, double-blind, controlled trial involving children aged 6 months to 18 years with acute asthma in the ED; Sung et al. investigated the clinical effects of nebulized budesonide [34]. All patients received oral prednisone at a dose of 1mg / kg and nebulized salbutamol at a dose of 0.15 mg / kg every 30 minutes for the first three nebulization and each hour for 4 h. The experimental group (*n* = 24) received a single dose of 2,000 μg (4 ml) of nebulized budesonide and the control group (*n* = 20) received a nebulization of four ml of physiological saline. The primary objective of the study was a two-point change in the Pulmonary Index Score (PIS) that was correlated with the predicted FEV1 and hospitalization rate in a previous study [37]. This study did not show a statistically significant difference between the two groups. However, a median time of 1 h was found in the budesonide group compared to 2 h in the control group (*p* = 0.06) to achieve clinical improvement (two point decrease in the PIS) and the number of hospitalized patients was higher in the control group than in the budesonide group (5 vs 2, *p* = 0.22). Analysis of the survival curve showed that the ED discharge time was shorter in the budesonide group (*p* = 0.02). In a recent study, Upham et al. did not find statistically significant difference in a randomized trial of 169 acute asthma patients aged two to 18 years distributed into two groups who received in addition to the administration of albuterol, ipratropium bromide and prednisone (2mg / Kg with a maximum of 60mg) either a single dose of 2 mg (8 ml) of nebulized budesonide or the same amount of nebulizing physiological saline [35].

### Limitations

Our study has some limitations. First, the study lacks power to demonstrate a difference in the effect of treatment between the two treatment groups if it exists. Second, randomization resulted into two groups comparable to baseline and to control secondary attrition bias at premature discontinuation of treatment we replaced the missing data with an adverse event and conducted an intention-to-treat. The two groups were treated in the same way and differed only in the type of treatment administrated. The hospitalization decision was also based on objective criteria defined in advance. Moreover, the absence of difference between the two treated groups does not differ from the results of the single study carried out in adults. However, many statistical tests have been done in this study inflating type I error. The clinical relevance of our results does not differ from other studies based on respiratory function assessment. PEF was used as the standard criterion for evaluating the efficacy of bronchodilators in acute asthma, but when it was used to evaluate the benefits of corticosteroid therapy it was not effective [38, 39]. In a meta-analysis that evaluated the effects of corticosteroids in acute asthma, Rowe et al. did not find a difference in the PEF between the corticosteroid group and the placebo group while they observed a decrease in hospitalizations, duration of visit to ED and relapses in the corticosteroid group [40].

## Conclusion

Considering its limited power, our study suggests that the association of nebulized budesonide with hydrocortisone hemisuccinate has no additional effect over the use of hydrocortisone alone in adults’ acute asthma managed in the ED.

## Supplementary Information

**Additional file 1: Additional Figure 1.** Evolution of the respiratory rate. *p < 0.05 vs baseline in both groups. RR: respiratory rate. **Additional Figure 2.** Evolution of the dyspnea scale. *p < 0.05vs previous evaluation in both groups. **Additional Figure 3.** Evolution of the heart rate. * p < 0.05 vs baseline in the control. **Additional Table 1.** Baseline clinical characteristics in the most severe patients. **Additional Table 2** Outcomes and side effects in the most severe patients.

**Additional file 2.**

**Additional file 3.**

**Additional file 4.**

**Additional file 5.**

## Data Availability

The datasets used and/or analyzed during the current study are available from the corresponding author upon reasonable request.

## Abbreviations

BDP: Beclomethasone dipropionate
ED: Emergency department
FEV1: Forced expiratory volume in 1 second
HCHS: Hydrocortisone hemisuccinate
HR: Heart rate
ICS: Inhaled corticosteroid
IQR: Interquartile range
PEF: Peak expiratory flow
PIS: Pulmonary Index Score
RR: Respiratory rate
SCS: Systemic corticosteroids
SD: Standard deviation
