# Correction: Nebulized budesonide combined with systemic corticosteroid vs systemic corticosteroid alone in acute severe asthma managed in the emergency department: a randomized controlled trial

**DOI:** 10.1186/s12873-022-00700-x

**Published:** 2022-08-03

**Authors:** Soudani Marghli, Chafiaa Bouhamed, Amira Sghaier, Nabil Chebbi, Insaf Dlala, Samia Bettout, Achref Belkacem, Sarra Kbaier, Nahla Jerbi, Abdelouahab Bellou

**Affiliations:** 1 Faculty of Medicine of Monastir, University of Monastir, 5019 Monastir, Tunisia; 2 Emergency Department, Research Unit «Douleur thoracique», UR17SP09, Tahar Sfar University Hospital, 5100 Mahdia, Tunisia; 3 Faculty of Medicine of Sousse, University of Sousse, 4002 Sousse, Tunisia; 4 Institute of Sciences in Emergency Medicine, Department of Emergency Medicine, Guangdong Provincial People’s Hospital, Guangdong Academy of Medical Sciences, Guangzhou, Guangdong Province China; 5 Department of Emergency Medicine, Wayne State University School of Medicine, Detroit, MI USA

**Correction: BMC Emerg Med 22, 134 (2022)**

**https://doi.org/10.1186/s12873-022-00691-9**

The original article [1] contained incorrect affiliations for co-author, Abdelouahab Bellou which have since been corrected.
