# Supplementary material for: Nebulized budesonide combined with systemic corticosteroid vs systemic corticosteroid alone in acute severe asthma managed in the emergency department: a randomized controlled trial
Source: BMC Emerg Med. 2022 Jul 23;22:134. doi: 10.1186/s12873-022-00691-9 (PMC9308286; doi:10.1186/s12873-022-00691-9)
Supplement: Supplementary file 1 — Additional file 1: Additional Figure 1. Evolution of the respiratory rate. *p < 0.05 vs baseline in both groups. RR: respiratory rate. Additional Figure 2. Evolution of the dyspnea scale. *p < 0.05vs previous evaluation in both groups. Additional Figure 3. Evolution of the heart rate. * p < 0.05 vs baseline in the control. Additional Table 1. Baseline clinical characteristics in the most severe patients. Additional Table 2 Outcomes and side effects in the most severe patients. [file 12873_2022_691_MOESM1_ESM.docx]

**Additional files**

**Additional Figure 1:** Evolution of the respiratory rate. *p<0.05 vs baseline in both groups. RR: respiratory rate.

**Additional Figure 2:** Evolution of the dyspnea scale. *p<0.05vs previous evaluation in both groups.

**Additional Figure 3:** Evolution of the heart rate. * p<0.05 vs baseline in the control

**Additional Table 1** Baseline clinical characteristics in the most severe patients

|  | **Budesonide**  **N= 6** | **Control**  **N= 9** | **P**p |
| --- | --- | --- | --- |
| Respiratory rate, breaths/min | 33±5 | 29±6 | 0.199 |
| SatO2, % | 92±7 | 94±3 | 0.428 |
| PEF, L/min | 93±58 | 112±35 | 0.440 |
| PEF, % of the predicted | 17±11 | 23±6 | 0.195 |
| Systolic arterial blood pressure, mmHg | 130±11 | 128±22 | 0.821 |
| Diastolic arterial blood pressure, mmHg | 73±08 | 71±12 | 0.693 |
| Heart rate, beats/min | 112±26 | 103±11 | 0.383 |

*All data are presented as number (percentage) or mean (±SD), β2: short-acting beta_2_-mimetics, h: hour, PEF: peak expiratory flow*

**Additional Table 2** Outcomes and side effects in the most severe patients

|  | **Budesonide**  **N= 6** | **Control**  **N= 9** | **Absolute risk reduction (95% CI)** |
| --- | --- | --- | --- |
| Hospitalization, n(%) | 4(67) | 3(33) | -34(-83 to 15) |
| Discharge criteria before 180 min, n (%) | 3(50) | 4(44) | -6(-59 to 47) |
| Side effects, n (%)  Palpitation, n  Tremor, n  Headache, n  Dry mouth, n | 1(17)  1  1  0  0 | 4(44)  4  2  0  1 | 27(-17 to 71) |

*All data are presented as number (percentage)*
